# Analysis of Sec61p and Ssh1p interactions in the ER membrane using the split-ubiquitin system

**DOI:** 10.1186/1471-2121-14-14

**Published:** 2013-03-11

**Authors:** Carol Harty, Karin Römisch

**Affiliations:** 1 Cambridge Institute for Medical Research, Hills Road, Cambridge, CB2 2XY, UK; 2 Current address: Sauder School of Business, Henry Angus Building, 2053 Main Mall, University of British Columbia, Vancouver, BC V6T 1Z2, Canada; 3 Department of Microbiology, Faculty of Biology, Saarland University, Campus A1.5, Saarbruecken, 66123, Germany

**Keywords:** Protein translocation, Endoplasmic Reticulum, Sec61 channel, Split-ubiquitin system

## Abstract

**Background:**

The split-ubiquitin system monitors interactions of transmembrane proteins in yeast. It is based on the formation of a quasi-native ubiquitin structure upon interaction of two proteins to which the N- and C-terminal halves of ubiquitin have been fused. In the system we use here ubiquitin formation leads to proteolytic cleavage liberating a transcription factor (PLV) from the C-ubiquitin (C) fusion protein which can then activate reporter genes. Generation of fusion proteins is, however, rife with problems, and particularly in transmembrane proteins often disturbs topology, structure and function.

**Results:**

We show that both the Sec61 protein which forms the principal protein translocation channel in the endoplasmic reticulum (ER) membrane, and its non-essential homologue, Ssh1p, when fused C-terminally to CPLV are inactive. In a heterozygous diploid Sec61-CPLV is present in protein translocation channels in the ER membrane without disturbing their function and displays a limited set of protein-protein interactions similar to those found for the wildtype protein using biochemical methods. Although its expression level is similar, Ssh1-CPLV interactions are less strong, and, in contrast to Sec61p, Ssh1p does not distinguish between Sbh1p and Sbh2p. We show that interactions can be monitored by reporter gene activity or directly by PLV cleavage, which is more sensitive, but leads to quantitatively different results.

**Conclusions:**

We conclude that the split-ubiquitin system we used here has high fidelity, but low sensitivity and is of limited use for detection of new, transient interactions with protein translocation channels in the ER membrane.

## Background

Sec61p is the core component of the protein translocation channel in the ER membrane, and its association with other proteins determines whether it functions in cotranslational or posttranslational secretory protein transport into the ER [1]. In association with proteasome subunits it is likely also involved in retrograde transport of proteins from the ER to the cytosol for degradation [2,3]. Sec61p has a homologue in yeast, Ssh1p, which is about 30% identical to Sec61p at the amino acid level and has similar membrane topology [4]. Ssh1p is involved only in cotranslational protein import to the ER [4-6]. Sec61p and two small, tail-anchored proteins, Sbh1p and Sss1p, form channels for cotranslational protein import into the ER [1]. Ssh1p forms channels with the homologue of Sbh1p, a protein called Sbh2p, and Sss1p [4]. In yeast Sec61 channels, but not Ssh1 channels, can also form heptameric Sec complexes with the Sec63 complex which is composed of Sec63p, Sec62p, Sec71p, and Sec72p [1]. The heptameric Sec complex mediates posttranslational protein import into the yeast ER [1]. In addition, a fraction of yeast Sec61 channels can be found in large complexes with proteasomes and the Hrd1 ubiquitin ligase in the ER membrane which are likely engaged in dislocation and degradation of misfolded secretory proteins [2,7].

**Figure 1:**
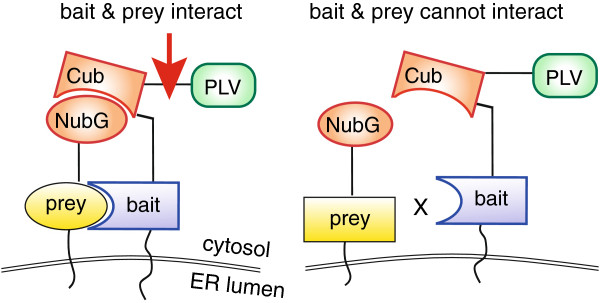
**The split-ubiquitin system.** The C-terminus of the protein of interest (bait) is fused to the C-terminal half of ubiquitin (Cub) followed by the PLV transcription factor. The N-terminal half of ubiquitin (Nub) is fused to the N- or C-terminus of a potential interactor (prey). Prey and bait fusion proteins are co-expressed in yeast with reporter genes HIS3 and LacZ under control of the LexA promoter. If prey and bait interact (left), Nub and Cub form a quasi-native ubiquitin structure which is recognized by cytosolic ubiquitin-specific proteases (red arrow). These cleave C-terminally of Cub and release PLV to activate transcription of HIS3 and LacZ. If prey and bait do not interact (right), Nub and Cub do not associate, PLV remains tethered to the bait, and HIS3 and LacZ remain silent.

All of the essential subunits of the protein translocation channel in the ER are integral membrane proteins which form dynamic complexes with the channel-forming subunits Sec61p and Ssh1p [1]. The split-ubiquitin system developed by Varshavsky and colleagues which allows monitoring even transient interactions of membrane proteins in intact cells seems ideally suited to investigate interactions of the core subunits of the ER translocation channels with accessory proteins in situ [8]. Ubiquitin is a 76 amino acid protein which can be conjugated to lysine residues of proteins with a variety of consequences, the best known being proteasomal degradation [9]. Proteins fused C-terminally to ubiquitin are cleaved from it by cytosolic ubiquitin-specific proteases (UBPs) [9]. In the split-ubiquitin system, the N-terminal half of ubiquitin (Nub; amino acids 1-37) is fused N- or C-terminally to a potential interactor (prey). The C-terminal half of ubiquitin (Cub; amino acids 35-76) is fused to the C-terminus of a protein of interest (bait) followed by a reporter. We employed the variation of the system developed by te Heesen and Stagljar [10,11]. Here Nub is modified by replacing the isoleucine at position 13 with glycine (NubG). NubG does not spontaneously associate with Cub. In this system the reporter following Cub is a 47 kDa transcription factor consisting of Protein A, the LexA DNA binding domain, and the transcriptional activation domain of VP16 (PLV). Bait and prey are expressed in a yeast strain with the reporter genes *HIS3* and *LacZ* under the LexA promoter. If bait and prey interact (Figure 1, left), NubG associates with Cub to form quasi-native ubiquitin. UBPs recognize this quasi-native ubiquitin and cleave and release PLV from the C-terminus of Cub. Cleaved PLV migrates to the nucleus and activates *HIS3*, which allows growth in the absence of histidine, and *LacZ* encoding beta-galactosidase which leads to blue colouring of cells growing on X-gal.

A different variant of the split-ubiquitin system has been used to demonstrate interactions of translocon components, but neither Sec61p nor Ssh1p were used as baits [6,12]. The CPLV-based version of the split-ubiquitin system has been used to investigate topology and interactions of oligosaccharyl transferase (OST) subunits [13-15]. OST is a large oligomeric complex of transmembrane proteins in the ER membrane which is responsible for N-glycosylation of nascent secretory proteins [16]. Since it is located in close proximity to protein translocation channels in the ER membrane, translocon interactions were also monitored by the authors directly [14,15]. In one instance, however, the authors used CPLV-fusions to Sbh1p and Sbh2p [14]. Both proteins are small, tail-anchored subunits of the Sec61 and the Ssh1 channel, respectively, with their C-termini in the ER lumen. Since positive interactions were found with the Sbh1-CPLV and Sbh2-CPLV fusion proteins, and the interacting Nub-fusions were located in the cytosol, adding CPLV to the C-terminus of Sbh1p and Sbh2p must have inverted their topology in the ER membrane, and it remains therefore unclear whether the interactions found with these fusion proteins are meaningful. Chavan et al. [15] also used a Sec61-CPLV construct to monitor translocon interactions, and the same construct was used later in a paper characterizing a *sec61* mutant [17]. The fusion protein in this case, however, was expressed ectopically from a plasmid in presence of a chromosomal wildtype *SEC61*. The functionality and expression level of the fusion protein was not investigated.

The Sec61p C-terminus is important for function and fusion to epitope tags or GFP has been shown to compromise Sec61 channel function to various degrees (Barrie Wilkinson, pers. communication; [18]; KR, unpublished). Here, we therefore asked whether Sec61p and Ssh1p fused to CPLV at their C-termini were functional and whether interactions found with these fusion proteins were physiologically meaningful.

## Results

### Sec61-CPLV and Ssh1-CPLV are dysfunctional

**Figure 2:**
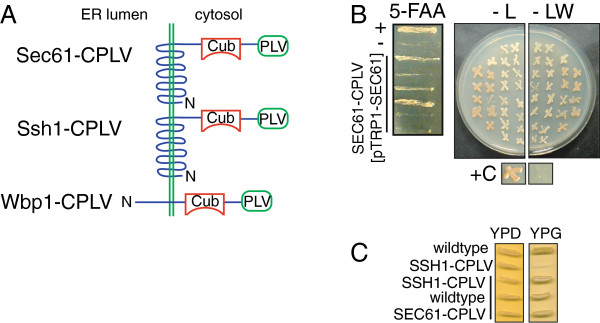
**Characterization of bait fusion proteins for the split-ubiquitin system. A**) Schematic representation of bait fusions in the ER membrane. **B**) Left: 5-FAA counterselection against a *SEC61*-*TRP1* plasmid in a *SEC61*-*CPLV* haploid. (+) positive control, strain carrying a non-essential *TRP1* plasmid; (-) negative control, strain is chromosomally *TRP1*. Right: Haploid *SEC61*-*CPLV* colonies that grew on 5-FAA were restreaked onto medium lacking leucine only (-L) or lacking both leucine and tryptophan (-LW); (+C) positive control, strain carrying a non-essential *TRP1* plasmid after growth on 5-FAA. **C**) Test for respiratory competence. Haploid wildtype and *SSH1*-*CPLV* yeast (top), and wildtype or heterozygous diploid (black bar) yeast were grown on full medium containing a fermentable (glucose, YPD) or non-fermentable (glycerol, YPG) carbon source.

In order to generate a strain in which interactions with Sec61p could be monitored using the split-ubiquitin system, we initially tried to integrate the *SEC61*-*CPLV* construct (Figure 2A) into the chromosomal *SEC61* locus of the haploid reporter strain L40 [10]. Since we were unable to do so, we then generated a diploid L40 derivative, in which one copy of *SEC61* was replaced with *SEC61*-*CPLV* on a *LEU2* integration plasmid. Sporulation and tetrad dissection always resulted in 2 live and 2 dead spores, the dead spores carrying the *SEC61*-*CPLV* construct. Since it was a possibility that germination rather than viability itself was primarily affected, we then transformed the *SEC61/SEC61*-*CPLV* heterozygous diploid with a plasmid containing wildtype *SEC61* and the *TRP1* auxotrophic marker. Upon sporulation and tetrad dissection of this strain all daughter cells survived, and two from each tetrad were able to grow on medium lacking leucine, suggesting that they contained the chromosomal *SEC61*-*CPLV* fusion. We then tried to counterselect against the *SEC61* plasmid in these cells using plates containing 5-fluoroanthranilic acid (5-FAA). This compound is converted to toxic 5-fluorotryptophan by cells which are prototrophic for tryptophan biosynthesis. The *SEC61*-*CPLV* haploid cells should only be able to grow on 5-FAA if they can lose the plasmid containing *TRP1* and *SEC61*. Initially we indeed found that several of the *SEC61*-*CPLV* haploid cells were able to grow on 5-FAA (Figure 2B, left). Restreaking of these cells on minimal medium lacking leucine and tryptophan, however, showed that all of them still were able to grow without tryptophan and still retained the *TRP1* plasmid with the *SEC61* gene (Figure 2B, right, -LW). In contrast, a control strain carrying a non-essential *TRP1* plasmid was able to lose it on 5-FAA and was subsequently no longer able to grow on plates lacking tryptophan (Figure 2B, bottom, compare -L and -LW). We conclude that the Sec61-CPLV fusion protein is dysfunctional and on its own does not support life of yeast cells.

The gene encoding the *SEC61* homologue *SSH1* is not essential, and we were able to replace the wildtype gene in the haploid L40 strain by integration of the *SSH1*-*CPLV* construct (Figure 2A). In some, but not all yeast strain backgrounds, cells with mutations in *SSH1* become respiration deficient and are no longer able to grow on non-fermentable carbon sources such as glycerol [5,6]. We found that our *SSH1*-*CPLV* haploid strain was unable to grow on glycerol indicating that the Ssh1-CPLV fusion protein was dysfunctional (Figure 2C, *SSH1*-*CPLV* haploid). We conclude that CPLV fusion to the C-termini of Sec61 and Ssh1p interferes with important interactions of these domains which are essential for protein function.

### Expression of *SEC61*-*CPLV* and *SSH1*-*CPLV* in presence of the wildtype genes has no effects on ER import or protein trafficking

**Figure 3:**
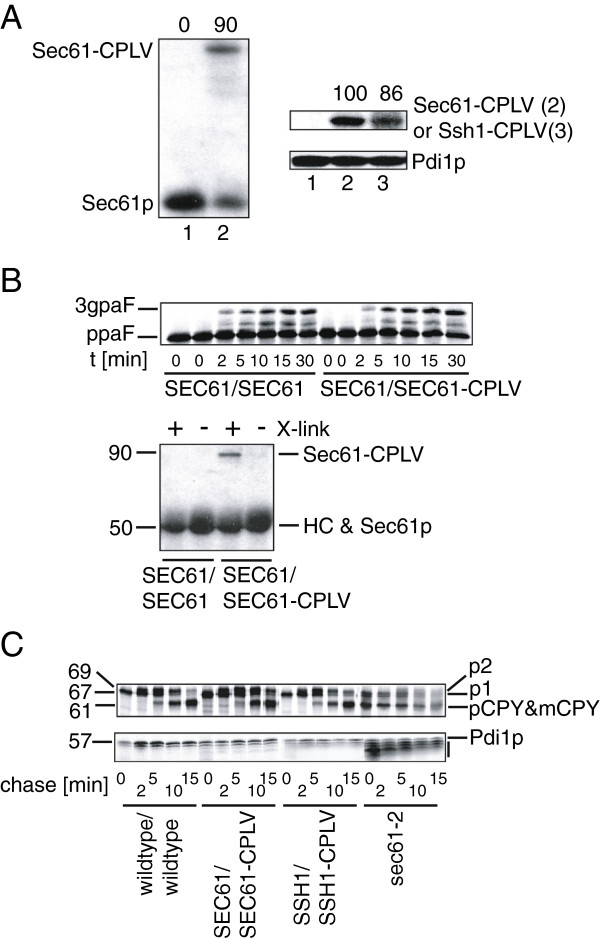
***SEC61*-*CPLV*and*SSH1*-*CPLV *do not affect ER import in heterozygous diploids. A**) Bait quantitation. Left: Wildtype (1) or *SEC61/SEC61*-*CPLV* yeast (2) were grown to early exponential phase, equal amounts lysed, proteins separated by SDS-PAGE, transferred to nitrocellulose, and detected with polyclonal anti-Sec61p-N- antiserum and ^125^I-Protein A. Right: Wildtype (1) or *SEC61/SEC61*-*CPLV* (2) or *SSH1/SSH1*-*CPLV* (3) cells were analyzed as above. Fusion proteins were detected with polyclonal anti-PLV-C-terminus, Pdi1p was a loading control. **B**) *SEC61-CPLV* does not affect posttranslational import into the ER, although associated with the Sec complex. Top: Import reactions containing microsomes from wildtype or *SEC61/SEC61-CPLV* diploid yeast, ^35^S-labelled preproalpha factor (ppaF), and ATP were incubated at 24°C for the indicated times, reactions stopped, and proteins detected by SDS-PAGE and autoradiography. ER-imported ppaF is signal-cleaved and glycosylated (3gpaF). Bottom: Import reactions as above were incubated at 20°C for 10 min, DMSO (-) or 500 μM DSP (+) was added for crosslinking. After quenching, membranes were lysed and Sec complex-associated proteins precipitated with polyclonal anti-Sec63p-serum. Crosslinks were reduced, proteins separated by SDS-PAGE and Sec61-CPLV and Sec61p detected with anti-Sec61p-N-terminus. **C**) ER import of CPY and Pdi1p is not affected in bait strains. Diploid cells expressing indicated fusion proteins and a *sec61*-*2* haploid with an ER protein import defect were grown to early log phase at 30°C, pulsed for 2 min with ^35^S-methionine/cysteine, and chased for indicated times. Cells were lysed, CPY (top) or Pdi1p (bottom) immunoprecipitated with polyclonal antisera, separated by SDS-PAGE and detected by autoradiography. Note that cytoplasmic precursor pCPY and mature vacuolar mCPY migrate at the same position. p1: ER CPY; p2: Golgi CPY. Black bar right of Pdi1p blot indicates hypoglycosylated forms of Pdi1p which occur when translocation is inefficient (in *sec61*-*2* cells).

Since both Sec61p and Ssh1p are large polytopic proteins which can bind to interacting proteins via domains other than the C-terminus, we asked whether we could use our CPLV-fusion proteins to monitor such interactions. In order for the results to be meaningful, the fusion proteins should be incorporated into functional translocons in the heterozygous diploids expressing both wildtype and CPLV fusion proteins. While the translocation pore for import into the ER may be formed by a single Sec61 complex, biochemical studies show that 2-4 Sec61 complexes are associated with ribosomes during translocation, and indeed this multimerization is triggered by the arrival of signal-sequence containing ribosome/nascent chain complexes at the ER membrane [19,20]. It was therefore reasonable to assume that Sec61-CPLV and Ssh1-CPLV would be incorporated into such complexes, if they were expressed at levels comparable to the wildtype proteins in the heterozygous diploid strains. Quantitative immunoblotting on extracts from the *SEC61/SEC61*-*CPLV* strain confirmed that expression of wildtype and fusion protein were approximately equal (Figure 3A, left). Since there are no antibodies against Ssh1p, we assessed expression of the fusion protein in the *SSH1/SSH1*-*CPLV* strain by comparing it to the amount of Sec61-CPLV in the heterozygous *SEC61/SEC61*-*CPLV* strain using an antibody against CPLV that we had raised and against Pdi1p as an internal marker, We found that expression levels of both fusion proteins were comparable (Figure 3A, right).

We next asked whether presence of the Sec61-CPLV protein had an effect on posttranslational import into the ER. Posttranslational import into the ER is highly sensitive to even relatively minor insults to the translocation channel, and can be monitored with maximum sensitivity using an in vitro assay based on isolated yeast ER membranes, and a radio-labelled, in vitro translated protein, preproalpha factor, which is posttranslationally imported [18,21]. When we compared a time course of import into membranes prepared from the *SEC61* wildtype diploid or those from the *SEC61/SEC61*-*CPLV* heterozygous diploid we found that there was no difference in import kinetics (Figure 3B, top). We then confirmed that the Sec61-CPLV fusion protein was present in posttranslational import channels in these membranes by crosslinking membrane proteins with the cleavable crosslinker dithiobissuccinimidyl propionate (DSP), immunoprecipitating with an antibody against Sec63p, and resolving immunoprecipitated proteins after cleavage of the crosslinker with dithiotreitol (DTT) on SDS gels. As shown in Figure 3B, bottom, Sec61-CPLV was clearly detectable in the Sec complexes from the heterozygous diploid strain.

In order to exclude any negative effects of the fusion proteins on ER-translocation or protein trafficking in general, we performed pulse-chase experiments in wildtype, *SEC61/SEC61*-*CPLV* and *SSH1/SSH1*-*CPLV* strains. Transit of the vacuolar protein carboxypeptidase Y (CPY) can be monitored by its molecular weight changes as it moves through the secretory pathway. As shown in Figure 3C, top, the kinetics of the appearance of the ER form (p1), the Golgi form (p2), and the mature vacuolar form (m) were identical in all three strains. In contrast, in a strain bearing the *sec61*-*2* mutation, the cytosolic precursor of CPY (pCPY) accumulated and appearance of the mature form was delayed (Figure 3C, top). Both CPY and preproalpha factor are imported into the ER posttranslationally. As Ssh1p is only involved in co-translational import, we performed a pulse-chase monitoring translocation of Pdi1p into the ER. Pdi1p is imported using both co- and posttranslational pathways, and defects in either lead to accumulation of the cytosolic precursor [22]. As shown in Figure 3C, bottom, there was no precursor accumulation in the *SSH1/SSH1*-*CPLV* strain. In contrast, cytosolic pPdi1p and underglycosylated forms due to delayed import into the ER were found in the translocation defective *sec61*-*2* mutant (Figure 3C, bottom). We conclude that Sec61-CPLV and Ssh1-CPLV do not interfere with import into the ER of heterozygous diploid strains.

### Detection of Sec61-CPLV and Ssh1-CPLV interactions in the ER membrane by growth and beta-galactosidase assays

**Figure 4:**
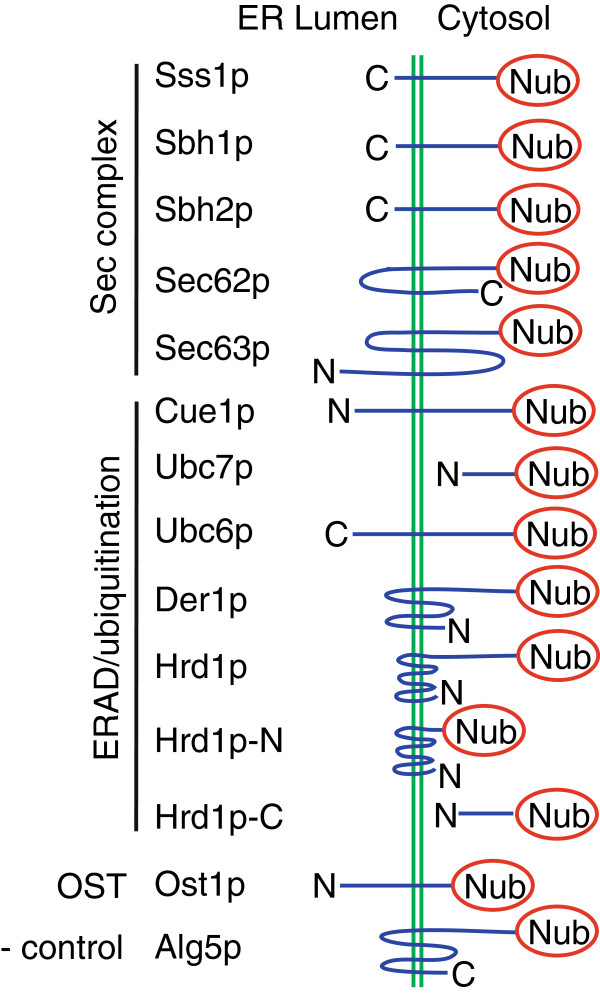
**Prey constructs for the split ubiquitin assay.** Proteins were fused as indicated to NubG. Topology of the fusion proteins in the ER membrane is shown. Interaction of Wbp1-CPLV with Ost1-Nub was used as positive control. Expression of Nub-Alg5 in the ER membrane was used as negative control for all bait constructs.

**Figure 5:**
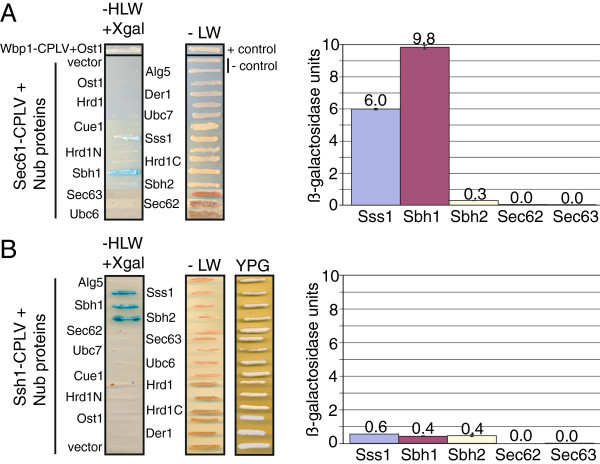
**Interactions of Sec61p and Ssh1p bait proteins in the split ubiquitin system.** Prey fusion proteins were expressed in heterozygous diploid *SEC61/SEC61*-*CPLV* (**A**) or heterozygous diploid *SSH1/SSH1*-*CPLV* yeast (**B**). Interactions were monitored by growth in the absence of histidine (-HLW) and ß-galactosidase activity leading to blue staining on Xgal plates (left) or measured in liquid ß-galactosidase assays (right). Expression of ALG5-Nub was used as negative control (- control), a haploid strain expressing *WBP1-CPLV* and *OST1*-*Nub* as positive control (+ control). Plates contaning histidine were used for growth control (-LW), and plates containing glycerol (YPG) to monitor growth on a non-fermentable carbon source for *SSH1-CPLV* strains. For liquid ß-galactosidase assays cells were grown to OD600 = 1.0, and activity measured as described in the Methods section. Values from six samples were averaged for each bait-prey combination.

Since Sec61-CPLV expressed in a *SEC61/SEC61*-*CPLV* heterozygous diploid was incorporated into Sec complexes (Figure 3B) we asked whether we could monitor other translocon-relevant protein-protein interactions using this construct and its homologue Ssh1-CPLV. We transformed the heterozygous diploid *SEC61/SEC61*-*CPLV* and *SSH1/SSH1*-*CPLV* strains with the bait constructs shown in Figure 4. The prey proteins we used included a negative control (Alg5p), Sec complex subunits (Sss1p, Sbh1p, Sbh2p, Sec62p, and Sec63p), proteins involved in polyubiquitination or ER export of proteins destined for ER-associated degradation (ERAD) (Hrd1p, Der1p, Cue1p, Ubc6p, and Ubc7p), and an oligosaccharyltransferase subunit (Ost1p) (Figure 4) [3]. Prey proteins were chosen for their localization to the ER membrane and for their potential for interaction with Sec61p and possibly Ssh1p. The gene for each prey protein was expressed in frame with the gene for Nub at its 3^′^ or 5^′^ end, such that Nub was located in the cytosol in the fusion protein. All prey constructs were expressed from 2-micron plasmids with the *TRP1* auxotrophic marker except *SEC63*-Nub, which was expressed from a CEN plasmid. Interaction of bait and prey proteins was detected by growth on minimal plates without histidine and expression of beta-galactosidase. The medium also lacked tryptophan to ensure maintenance of the prey protein plasmids and leucine to guard against loss of the integrated bait construct. The positive control was the haploid standard yeast strain used for the split-ubiquitin system (L40) co-expressing Ost1p-Nub and Wbp1-CPLV from plasmids. These cells grew in the absence of histidine, and beta-galactosidase activity was visible on media containing X-gal (light blue colour, Figure 5A, top). In the *SEC61/SEC61*-*CPLV* and *SSH1/SSH1*-*CPLV* heterozygous diploids Nub-Alg5p, which is an ER-membrane protein not expected to interact with the translocon, served as a negative control. Neither NubG-Alg5p nor empty vector enabled growth on medium lacking histidine when co-expressed with Sec61-CPLV or Ssh1-CPLV (Figure 5). For the *SEC61/SEC61*-*CPLV* heterozygous diploid, only cells expressing Nub-Sss1p or Nub-Sbh1p or (to a lesser extent) cells expressing Nub-Sbh2p grew, and had beta-galactosidase activity after 3 days of incubation at 30°C (Figure 5A). No growth was observed of cells expressing Nub-fusions to other translocon subunits (Sec62p and Sec63p) or to the ERAD-relevant proteins (Figure 5A). This suggests either that there was no interaction between these fusion proteins and Sec61p-CPLV, or that the interactions were too weak to allow sufficient cleavage of PLV to induce the reporter genes.

With the *SSH1/SSH1*-*CPLV* heterozygous diploid, growth and beta-galactosidase activity were also only observed for cells expressing Nub-Sss1p, Nub-Sbh1p, and Nub-Sbh2p after 3 days of incubation at 30°C (Figure 5B). Strikingly, whereas Sec61-CPLV seemed to be able to distinguish between Sbh1p and Sbh2p, Ssh1-CPLV interacted equally strongly with both Nub fusion proteins (compare Figure 5A and B). Again no growth or activity was observed for cells expressing the Nub-fusions to other translocon subunits (Sec62p and Sec63p) or to ERAD-relevant proteins (Figure 5B). These results suggest that both Sec61p-CPLV and Ssh1p-CPLV interact closely with Nub-Sss1p, Nub-Sbh1p, and Nub-Sbh2p, but do not interact with the other prey fusion proteins closely or strongly enough for Nub and Cub to associate.

In order to be able to compare the relative strenghts of the associations we also performed beta-galactosidase assays on extracts from the strains expressing the various bait and prey fusion proteins. Lysates from 0.3 OD_600_ cells were suspended in buffer containing ONPG and beta-mercaptoethanol, and incubated at 30°C for 180 minutes. The reaction was stopped by adding Na_2_CO_3_, and the OD_420_ of the supernatant was measured. Averaged measurements from 6 samples were used to calculate beta-galactosidase units. As shown in Figure 5, right, in this assay Sec61-CPLV interacted strongly with Nub-Sss1p (6.0 U) and nearly twice as strongly with Nub-Sbh1p (9.8 U), but only weakly with Nub-Sbh2p (0.28 U). These measurements were consistent with cell growth and beta-galactosidase activity seen on plates (Figure 5A, left). Despite expression levels similar to Sec61-CPLV (Figure 3A), Ssh1-CPLV interacted about tenfold more weakly with Nub-Sss1p (0.55 U) and about twentyfold more weakly with Nub-Sbh1p (0.43 U), but about twofold more strongly with Nub-Sbh2p (0.45 U) (Figure 5, right panels). It was possible that the differences in the strengths of response in the split-ubiquitin system between the *SEC61/SEC61*-*CPLV* heterozygous diploid and the *SSH1/SSH1*-*CPLV* heterozygous diploid resulted from different expression levels of prey fusion proteins rather than differences in the strengths of the actual bait-prey interactions, but we found that Nub-Sss1p and Nub-Sbh1p were both expressed in the *SEC61/SEC61*-*CPLV* heterozygous diploid and the *SSH1/SSH1*-*CPLV* heterozygous diploid at similar levels (not shown). This suggests that the differences in prey interaction strengths between Sec61p-CPLV and Ssh1p-CPLV were due to genuine differences in the strength of the interactions. Nub-Sec62p was also expressed similarly in the *SEC61/SEC61*-*CPLV* heterozygous diploid and the *SSH1/SSH1*-*CPLV* heterozygous diploid and at levels comparable to the wildtype protein (not shown). Using the quantitative liquid beta-galactosidase assay there was no detectable interaction of either Sec61p-CPLV or Sshp1-CPLV with Nub-Sec62p, however, or with any of the other prey fusion proteins (not shown). This was consistent with the results of the split-ubiquitin plate assay.

### Detection of Sec61-CPLV and Ssh1-CPLV interactions in the ER membrane via proteolytic cleavage of PLV

**Figure 6:**
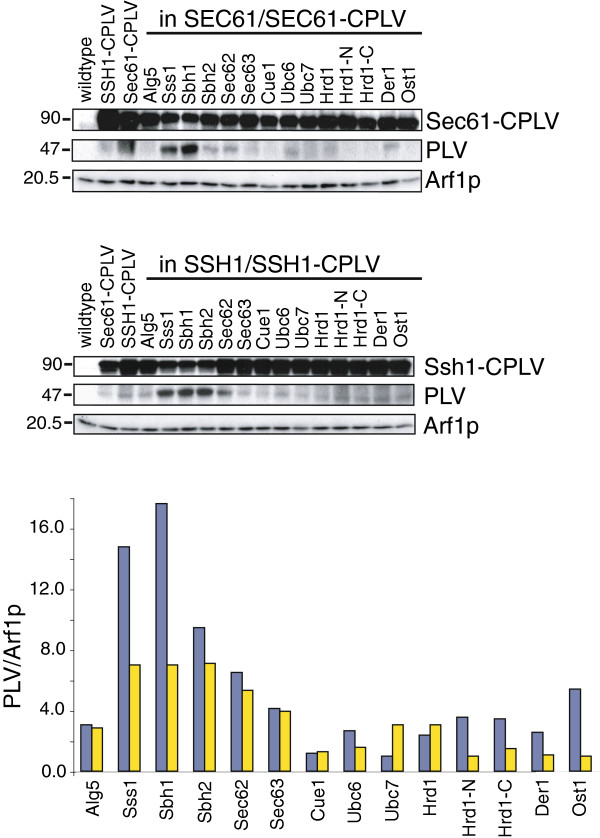
**Interactions of Sec61p and Ssh1p bait proteins using PLV cleavage as readout.** Prey proteins were expressed in heterozygous diploids expressing the bait-fusion proteins as above. Cells were grown to early exponential phase in minimal medium, and lysates of 1 OD600 were separated by SDS-PAGE, proteins transferred to nitrocellulose, and quantitative immunoblotting performed with polyclonal rabbit antibodies against PLV, to detect intact and cleaved bait proteins, and Arf1p (as loading control) and ^125^I-Protein A. The ratios of cleaved PLV to Arf1p for each strain are shown in the graph in blue for Sec61-CPLV and yellow for Ssh1-CPLV.

As we were unable to detect some of the expected interactions of Sec61p (e.g. with Sec62p) using either the plate assay or the liquid beta-galactosidase assay, it seemed possible that some bait-prey interactions released some, but not sufficient PLV to reach the threshold of reporter gene activation. We therefore also directly monitored cleavage of PLV from the bait fusion proteins. We raised a polyclonal antibody against PLV and performed quantitative immunoblots on cell lysates from log phase cultures of bait strains co-expressing prey fusion proteins. As shown in Figure 6, when Sec61-CPLV was co-expressed with prey proteins previously shown to interact with Sec61-CPLV, a band appeared at 47 kDa, corresponding to the cleaved PLV fragment concomitant with a decrease in intensity of the Sec61-CPLV band (Figure 6, top). The cleaved PLV fragment was strongest for cells co-expressing Nub-Sss1p and Nub-Sbh1p with Sec61-CPLV (Figure 6, top). The band also appeared more faintly in lysates from cells co-expressing Sec61-CPLV and Nub-Sbh2p, Nub-Sec62p, Ubc6p-Nub, Hrd1p-Nub, and Der1p-Nub, and very faintly for Sec63p-Nub. We quantified the cleaved PLV with respect to Arf1p (Figure 6, bottom, blue bars). Quantitation showed that only co-expression of Sec61-CPLV with Nub-Sss1p, Nub-Sbh1p, Nub-Sbh2p, Nub-Sec62p and possibly Ost1p-Nub resulted in substantially (approximately twofold) more cleavage of PLV than the negative control Nub-Alg5p. For the *SSH1/SSH1*-*CPLV* heterozygous diploid, appearance of the 47 kDa cleavage product and reduction of intensity of the Ssh1-CPLV band indicated that there was cleavage of the bait fusion protein in cells co-expressing Ssh1-CPLV with Nub-Sss1p, Nub-Sbh1p, and Nub-Sbh2p, and probably Nub-Sec62p as well (Figure 6, middle). Quantitation showed that only co-expression of Ssh1-CPLV and these 4 prey proteins resulted in substantially (approximately twofold) more cleavage of PLV than with the negative control Nub-Alg5p (Figure 6, bottom, yellow bars).

In the *SEC61/SEC61*-*CPLV* heterozygous diploid the relative intensity of cleaved PLV (expressed in arbitrary units) corresponded to some degree to the beta-galactosidase activity shown in Figure 5. For Sec61-CPLV interactions, the largest amounts of PLV cleavage product were produced by expressing Nub-Sbh1p (18), Nub-Sss1p (15), and Nub-Sbh2p (9.6), followed by Nub-Sec62p (6.6) and Ost1p-Nub (5.5), compared to the negative control Nub-Alg5p (3.1) (Figure 6, bottom, blue bars). For the *SSH1/SSH1*-*CPLV* heterozygous diploid, the strongest PLV cleavage signals were with Nub-Sbh2p (7.2), Nub-Sss1p (7.1), Nub-Sbh1p (7.1), and Nub-Sec62p (5.4), compared to the negative control Nub-Alg5p (2.9) (Figure 6, bottom, yellow bars). The relationship of PLV cleavage with gene activation, however, proved to be non-linear. For example, the difference between Ssh1-CPLV interaction with Nub-Sbh1p and Sec61-CPLV interaction with Nub-Sbh1p is about 20× in the beta-galactosidase liquid assay, but only 2.5× as measured by PLV cleavage (compare Figure 5, right, magenta bars, with Figure 6, bottom, blue vs. yellow bars). The threshold for reporter gene activation by PLV cleavage was approximately 6, as measured by immunoblotting (compare Figure 6, bottom to Figure 5). We conclude that PLV cleavage is a more sensitive readout for protein interaction in this version of the split-ubiquitin system than reporter gene activation.

## Discussion

### Characterization of Sec61-CPLV and Ssh1-CPLV proteins

Interactions between transmembrane proteins are notoriously difficult to study using biochemical methods thus the split-ubiquitin system with its ability to detect even transient interactions in situ had unique potential to explore interactions with the protein translocation channel core subunits in the ER membrane. Here we asked whether we could study interactions of the essential translocon subunit Sec61p and its nonessential homologue Ssh1p by using either as bait in the split-ubiquitin system. Initially, we tried to create strains in which *SEC61*-*CPLV* or *SSH1*-*CPLV* replaced the respective wildtype genes. This would have ensured that the bait fusion proteins were incorporated into functional translocons, increasing confidence that any interactions detected were physiological. Our attempts to generate a viable haploid *SEC61*-*CPLV* strain failed, however, in spite of using a variety of techniques (Figure 2). The haploid *SSH1*-*CPLV* strain was viable, but respiration deficient (Figure 2C). Wilkinson *et al*. [5] had reported that *ssh1Δ* cells are viable but respiration deficient and that respiration deficiency reduces the load on the secretory pathway, thus compensating for loss of function of Ssh1p. Our finding that the *SSH1*-*CPLV* haploid is respiration deficient indicates that Ssh1p-CPLV lacks one or more functions of wildtype Ssh1p, and that this loss of function stresses the secretory pathway sufficiently to allow spontaneously occurring respiration deficient cells to have a growth advantage.

Fusions to *SEC61* have been shown previously to compromise Sec61p function to various degrees: an early C-terminal GFP fusion was viable only when overexpressed, a His6-tag at either N- or C-terminus reduces the speed of protein translocation through the channel into the ER, and a C-terminal 13 myc tag interferes with posttranslational import into the ER (B. Wilkinson, pers. communication [18,23]). Adding a variety of GFP variants and other tags to the Sec61p C-terminus via a yeast codon-optimized 8 amino acid linker, however, seems to not grossly affect viability, but protein translocation was not examined specifically in these cells [24]. Wittke *et al*., [12] used Sec61p as prey in their version of the split ubiquitin system by fusing Nub to the Sec61p N-terminus and replacing the chromosomal wildtype *SEC61*. Cells expressing solely Nub-Sec61p were viable, but effects on protein import into the ER were not examined specifically [12]. Sec61p has also previously been used as split-ubiquitin bait fused to CPLV at the C-terminus, but in this work the protein was expressed ectopically from a plasmid in presence of the wildtype protein, and its functionality was not examined [14,17]. The functions of our Sec61-CPLV and Ssh1-CPLV proteins might be compromised due to the large size of the tag (47 kDa) or its position at the C-terminus. The addition of a 13myc tag, which is comparable in size to CPLV, to the Sec61p C-terminus, however, resulted in viable cells with primarily posttranslational import defects [18]; KR, unpublished). This suggests that the Sec61p C-terminus might be important for productive interactions with the Sec63 complex. The structure of a channel homologous to the Sec61 channel, the *E*. *coli SecYEG* channel, docked to a ribosome-nascent chain complex revealed that the C-terminus of the SecY protein actually reaches into the polypeptide exit tunnel of the ribosomal large subunit [25]. Since some fusions to the yeast Sec61 C-terminus are viable, its interaction with the ribosome is likely not essential in yeast, but if the linker used is not sufficiently flexible, a bulky tag might interfere with ribosome/Sec61 channel interactions, or Sec63 complex/Sec61 channel interactions. The linker that we used between Sec61p and CPLV was a 9 amino acid peptide derived from the vector sequence (ESGGSTMSG). It was not that different in size or composition from the linker used for the GFP fusion cassettes in Young *et al*. [24] (GDGAGLIN) which resulted in viable transformants when fused to chromosomal *SEC61*, but in contrast to the latter neither our linker nor our tag were codon-optimized for yeast. We did, however, see approximately equal expression of Sec61p and Sec61-CPLV in our heterozygous diploid cells (Figure 3A), suggesting that the defects in the fusion proteins were due to direct interference of the tag with Sec61p and Ssh1p function, and not simply due to low expression or protein instability.

### Prey interactions with Sec61-CPLV and Ssh1-CPLV

Although we had shown that Sec61p-CPLV and Ssh1p-CPLV were dysfunctional, they might still have been suitable to identify protein translocation channel interactors in heterozygous diploid yeast, provided the fusion proteins were associated with functional protein translocation channels and did not interfere with translocation into the ER. We therefore established that heterozygous diploid strains expressing both bait and wildtype proteins were ER translocation and respiration-competent, and that Sec61-CPLV was associated with other Sec complex subunits in the ER membrane (Figures 2, 3).

When we transformed the *SEC61/SEC61*-*CPLV* and the *SSH1/SSH1*-*CPLV* heterozygous diploids with constructs expressing prey proteins (Figure 4) and streaked cells co-expressing each bait-prey combination onto media lacking histidine and containing X-gal, we found a limited number of interactions: Nub-Sss1p, Nub-Sbh1p, and Nub-Sbh2p led to growth and blue colouring of cells expressing Sec61-CPLV or Ssh1-CPLV (Figure 5). The interaction of Ssh1-CPLV with Nub-Sbh1p was unexpected since the purified Ssh1 complex does not contain Sbh1p [4]. Expression levels of Nub-Sbh1p were similar to endogenous untagged Sbh1p (not shown), so overexpression did probably not contribute to this interaction. A more likely explanation might be that transient mispairing of Ssh1-CPLV with Nub-Sbh1p in the ER membrane was stabilized by the Nub-Cub interaction.

We detected no interactions of Sec61-CPLV or Ssh1-CPLV with subunits of the Sec63 complex or proteins involved in ubiquitination or ERAD (Figure 5), although we had been able to crosslink Sec61-CPLV to Sec63p (Figure 3B). The lack of interaction that we observed here may have resulted from the fact that the Sec61p C-terminus, to which CPLV is fused, is relatively short whereas the Sec63p C-terminus, to which Nub is fused, is much longer, so the two halves of ubiquitin may not have been able to interact due to their tethering to the ER membrane at substantially different distances. We noticed, however, that cells co-expressing Nub-Sec62p and Sec61-CPLV, although they did not grow, turned pale blue on medium lacking histidine and containing X-gal (not shown). We also observed that all cells expressing bait and prey combinations that interact turned blue prior to significant growth on plates (not shown). This suggested that induction of *lacZ* by cleaved PLV might have a lower threshold of induction than *HIS3*, and that a quantitative beta-galactosidase assay on cell lysates might therefore be more sensitive and detect weaker interactions than the growth assay. This turned out not to be the case: As for the growth assay shown in Figure 5, left, the only bait-prey combinations with significant beta-galactosidase activity in cell extracts were Nub-Sss1p, Nub-Sbh1p, and Nub-Sbh2p with either Sec61-CPLV or Ssh1-CPLV (Figure 5, right). The liquid beta-galactosidase assay did show, however, that the strength of the interactions differed substantially between Sec61-CPLV and Ssh1-CPLV: For Ssh1p-CPLV interactions of similar strengths were detected when expressing Nub fusions to Sss1p, Sbh2p, and Sbh1p (Figure 5, right). For Sss1p and Sbh1p, the interactions with Sec61-CPLV were 10- and 20-fold stronger than with Ssh1p (Figure 5, right), despite the fact that the expression levels of the bait fusion proteins were comparable (Figure 3A, right). Interactions of Sbh2p with Ssh1-CPLV and Sec61-CPLV were similar and relatively weak. These data suggest that Sec61p can distinguish between Sbh1p and Sbh2p which are 50% identical at the amino acid level [26]. One important difference between the two proteins is the presence of a phospho-threonine at position 5 in Sbh1p [27]. This phosphorylation site is conserved in mammalian Sec61beta, but absent in Sbh2p [27]. Whether phosphorylation of T5 affects Sbh1p interactions with Sec61p remains to be investigated. Despite the differential interaction of Sec61p with Sbh1p and Sbh2p, however, the presence of Sbh2p on its own is sufficient for Sec61 complex function [26]. So either Sbh2p forms complexes with Sec61p more readily in the absence of Sbh1p, or Sbh1p performs a regulatory function during translocation that Sbh2p can still fulfil when present in substoichiometric amounts [28].

We also directly monitored the appearance of the PLV cleavage product upon interaction of bait and prey by immunoblotting with polyclonal antibodies that we had raised against the cleavage product (Figure 6). We initially tried to express the amount of cleaved PLV as percentage of the total Sec61-CPLV or Ssh1-CPLV on the blot, but found that this resulted in reproducible numbers only where strong cleavage had occurred. Since the bait fusions were integrated into the chromosome and expressed from their own promoters at constant levels, and expression of the prey fusion proteins had no detectable effects on bait expression, we resorted to using the cytosolic protein Arf1p as a loading marker and measured the ratio of PLV/Arf1p in each strain instead. Using this readout, in addition to the interactions with Sss1p, Sbh1p, and Sbh2p with Sec61-CPLV we found cleavage of the fusion protein in the presence of Nub-Sec62p and Ost1p-Nub (Figure 6, top). Interactions of Sec61-CPLV with OST subunits had also been reported by the Lennarz group [15], and we had shown previously with the OST subunit Wbp1p as a bait and the translocon subunit Sss1p as prey that OST can interact with the Sec61 channel [21]. The immunoblot for PLV cleavage also indicates possible interaction of Ssh1p-CPLV with NubG-Sec62p, although no interactions of Ssh1p with Sec63 complex subunits have been observed using biochemical means (Figure 6, middle [4,5]). Whether this interaction is physiologically meaningful remains to be seen. The essential subunits of the Sec63 complex, Sec62p and Sec63p, both have cytosolic C-termini. A different version of the split-ubiquitin system has been used to demonstrate interaction of Sec63p bait with several prey fusions including NubG-Sec61p [12]. Sec62p has only been associated with posttranslational transport, while several reports suggest that Sec63p is required to recruit BiP to the ER membrane during both cotranslational and posttranslational import [29-31]. In mammalian cells Sec62p and Sec63p seem to have functions entirely separate from each other [32,33]. It might therefore be interesting to compare Sec63p-CPLV interactions against Sec62p-CPLV interactions with various prey proteins including Nub-Ssh1p and Nub-Sec61p, and to include the mammalian orthologues in this analysis.

## Conclusions

We have shown here that the Sec61-CPLV and Ssh1-CPLV bait fusion proteins for the split-ubiquitin system developed by te Heesen and Stagljar are dysfunctional. Characterizing the assay we demonstrated that monitoring PLV cleavage directly by immunoblotting is more sensitive than monitoring reporter gene activation, and that the relationship between PLV cleavage and reporter gene activation is non-linear in our system (compare graphs in Figure 5 to graph in Figure 6) [10,11]. Whereas another version of the split-ubiquitin system detects very transient interactions [8,34], in the system that we used reporter gene activation could only detect interactors that can also readily be crosslinked to or co-immunoprecipitated with the bait proteins, suggesting high fidelity but low sensitivity of the NubG/CPLV-based split-ubiquitin system.

## Methods

### Yeast strains & growth

Yeast strains used in this study are shown Table 1. Standard yeast media and growth conditions were used [35]. For counterselection of *TRP1* plasmids, yeast were streaked onto 5-FAA plates (0.5 g/l 5-fluoroanthranilic acid, Fluka, 5% glucose, appropriate supplements, 2% Bacto agar, 0.7% yeast nitrogen base) and incubated for 2-3 days at 30°C [36]. 1 OD_600_ represents 2.7 × 10^7^ cells for diploid yeast and 2.8 × 10^7^ cells for haploid yeast.

**Table 1:** ***S. cerevisiae *strains used in this study**

**Name**	**Genotype**	**Reference**
RSY255	*MATα leu2-3,112 ura3-52*	[23]
L40	*MATa trp1 leu2 his3 LYS2::lexA-HIS3*	[10]
	*URA3::lexA-LacZ*	
KRY411	*MATα trp1 leu2 his3 LYS2::lexA-HIS3*	[10]
	*URA3::lexA-LacZ*	
KRY511	*trp1 leu2 his3 LYS2::lexA-HIS3*	this study
	*URA3::lexA-LacZ*	
KRY518	*trp1 leu2 his3 LYS2::lexA-HIS3*	this study
	*URA3::lexA-LacZ LEU2::SEC61-CPLV*	
KRY548	*trp1 leu2 his3 LYS2::lexA-HIS3*	this study
	*URA3::lexA-LacZ SSH1 LEU2::SSH1-CPLV*	
KRY436	*MATa trp1 leu2 his3 LYS2::lexA-HIS3*	this study
	*URA3::lexA-LacZ LEU2::SSH1-CPLV*	

### Constructs

Nub fusions were cloned in frame with the respective genes into pRS314-NubG as described in Stagljar et al. [10], and Scheper et al. [21]. *SEC61*-*CPLV* and *SSH1*-*CPLV* were generated by replacing the *wbp1* fragment in pRS305(Δwbp1-Cub-PLV) with 5' truncated fragments of *SEC61* (missing the 5' 160 bp) or *SSH1* (missing the 5' 134 bp). Inserts were inserted into the XhoI site of the vector. For integration, plasmids were linearized within the *SEC61* and *SSH1* coding regions and integration into the correct chromosomal locus verified by PCR on chromosomal DNA. In the protein, the vector adds the amino acids ESGGSTMSG to the Sec61p/Ssh1p C-terminus before the PLV sequence described in [10].

### Pulse-chase experiments

Pulse chase experiments were performed as previously described [37]. Cells were grown to OD_600_ of 0.5-1.0 in minimal medium with the appropriate supplements, washed twice in labelling medium ((0.7% YNB without amino acids or ammonium sulphate (Difco), supplements appropriate for the auxotrophies of the strains used, and 5% glucose), preiincubated 30°C, 10 min, and pulsed with 0.35 mCi/ml Promix (Amersham) and incubated at 30°C, 2 or 5 min. To terminate labelling 250 μl 2× chase mix (0.6 mg/ml cysteine, 0.8 mg/ml methionine, 2.6 mg/ml ammonium sulphate, 200 mg/ml casamino acids (Difco) in labelling medium) was added. Cells were lysed by bead-beating, and proteins immunoprecipitated, separated by SDS-PAGE and detected by autoradiography.

### Co-immunoprecipitations, in vitro translocation, & Immunoblotting

Co-immunoprecipitation, in vitro translocation of preproalpha factor and immunoblotting was done as described in [21]. Proteins detected with antibodies against the Sec61p N-terminus (our lab), Pdi1p (our lab), CPY (gift from Randy Schekman), Arf1p (gift from Rainer Duden), or a polyclonal rabbit antibody that we raised for this work against the C-terminal 15 amino acids of PLV.

### Split-ubiquitin assays

#### Plate assay

Co-expression of bait and prey fusion proteins in KRY518 or KRY548 expressing NubG prey protein from a plasmid and streaking the cells onto minimal medium w/o leucine and tryptophan and onto X-gal plates lacking leucine, tryptophan, and histidine. A positive result is indicated by beta-galactosidase activity resulting in blue colouring on X-gal and by growth on medium lacking histidine [8,10].

#### Liquid beta-Galactosidase assay

Cells were grown in minimal medium lacking leucine and tryptophan at 30°C overnight to OD_546_ of 0.9-1.3, aliquots of 1 OD_546_ were centrifuged and washed in 1 ml buffer Z (113 mM Na_2_HPO_4_, 40 mM NaH_2_PO_4_, 10 mM KCl, and 1 mM MgSO_4_), and pellets were stored at –20°C. To perform the assay, 300 μl buffer Z was added to the pellets, vortexed and lysed by 3 freeze-thaw cycles. 100 μl lysate was immediately added to 700 μl buffer Z containing 0.27% beta-mercaptoethanol. 160 μl 4 mg/ml 2-nitrophenyl-beta-D-galactopyranoside (ONPG) in buffer Z was added and samples incubated at 30°C for 180 min. Reactions were stopped by adding 0.4 ml of 0.1 M Na_2_CO_3_, samples were centrifuged and the OD_420_ of the supernatant was measured. For each prey-bait combination 6 samples were taken and the results averaged.

$$\mathrm{beta}-\mathrm{galactosidase}\;\mathrm{units}=1000\times \left[{\mathrm{OD}}_{420}/\left({\mathrm{OD}}_{546}\times \mathrm{minutes}\;\mathrm{incubated}\right)\right]$$

## Competing interests

The authors declare no competing interests related to this article.

## Authors’ contributions

Both authors conceived the study and designed the experiments. CH carried out the research. KR wrote the manuscript with contributions from CH. Both authors read and approved the final manuscript.
